# AN AGE-RELATED POSITIVITY EFFECT IN HUMOR: AN ASIAN CONTEXT

**DOI:** 10.1093/geroni/igac059.786

**Published:** 2022-12-20

**Authors:** Zoe Ziyi Ng, W Quin Yow

**Affiliations:** Raffles Institution, Singapore, Singapore; Singapore University of Technology & Design, Singapore, Singapore

## Abstract

Humor plays a pivotal role in our interaction with people. According to the age-related positivity effect, older adults (OA) demonstrate more positive emotions and are better able to modulate negative emotions than younger adults (YA), suggesting an increase in humor appreciation with age. However, given the heterogeneity and complexity of humor, it is unknown if this effect holds across various types of humor in a culture with ambivalent attitudes toward humor (Yue, 2016). We explored age-related effects in the perception of four types of humor with Asian adults: incongruity-resolution/benign humor, aggressive humor, local-slang humor, and self-vs.-other-deprecating humor. Fifty-six Singaporean-Chinese OA (n=26;M=64.33) and YA (n=30;M=22.48) watched four short videos depicting the four types of humor in a randomized order. Participants rated how funny each video was (from a scale of 1-not funny-at-all to 5-very-funny), ranked them in the order of most-to-least-entertaining and answered questions on Future-Time-Perspective-scale and self-rated humor.Repeated-measures ANOVA found significant main effects of age, F(1,59)=6.60, p=.013, where OA gave higher ratings than YA (M=3.00 vs. 2.47), and humor-type, F(3,177)=26.87, p<.001. There was a significant linear contrast in humor-type where aggressive and benign humor had the lowest ratings while self-vs.-others-deprecating humor had the highest ratings. Ranking analyses revealed that OA preferred local-slang and others-deprecating humor while YA preferred other-deprecating humor significantly above chance. Interestingly, there was no relationship between self-rated humor and future time perspective with age. Overall, OA displayed a greater appreciation of humor than YA, supporting an age-related positivity effect in an Asian context, despite preference differences. We would like to thank Prof Suzanne Flynn, Department of Linguistics and Philosophy, MIT, for her guidance, and the MOE Tier 2 Academic Research Fund Tier 2 (T2MOE2005) and SUTD Growth Plan Grant for Healthcare (SGPHCRS1902) awarded to the last author.

